# Chlorido[1-(pyridin-2-yl)ethanone oximato-κ^2^ *N*,*N*′][1-(2-pyrid­yl)ethanone oxime-κ^2^ *N*,*N*′]copper(II) trihydrate

**DOI:** 10.1107/S1600536811049129

**Published:** 2011-11-23

**Authors:** Xiumin Qiu, Leilei Li, Dacheng Li

**Affiliations:** a School of Chemistry and Chemical Engineering, Liaocheng University, Shandong 252059, People’s Republic of China

## Abstract

In the title compound, [Cu(C_7_H_7_N_2_O)Cl(C_7_H_8_N_2_O)]·3H_2_O, the metal ion is five-coordinated by the N atoms from the 1-(pyridin-2-yl)ethanone oximate and 1-(pyridin-2-yl)ethanone oxime ligands and by the chloride anion in a distorted square-pyramidal geometry. The distortion parameter is 0.192. The two organic ligands are linked by an intra­molecular O—H⋯O hydrogen bond. In the crystal, mol­ecules are linked by O—H⋯O and O—H⋯Cl hydrogen bonds. The title compound is the hydrated form of a previously reported structure [Wu & Wu (2008 ▶). *Acta Cryst.* E**64**, m828]. There are only slight variations in the mol­ecular geometries of the two compounds.

## Related literature

For uses of oximes, see: Chaudhuri (2003 ▶). For theoretical research, see: Pavlishchuk *et al.* (2003 ▶). For related structure, see: Zuo *et al.* (2007 ▶); Wu & Wu (2008 ▶). For the properties of related complexes, see: Davidson *et al.* (2007 ▶); Clerac *et al.* (2002 ▶). For the distortion parameter, see: Addison *et al.* (1984 ▶). 
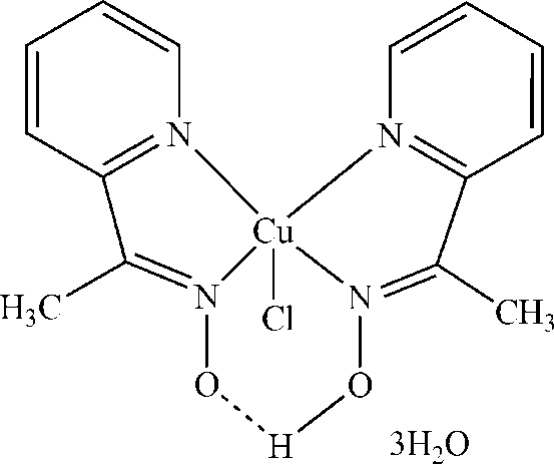


## Experimental

### 

#### Crystal data

[Cu(C_7_H_7_N_2_O)Cl(C_7_H_8_N_2_O)]·3H_2_O
*M* *_r_* = 424.34
Triclinic, 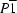

*a* = 8.3980 (9) Å
*b* = 10.2559 (11) Å
*c* = 12.1121 (13) Å
α = 114.199 (2)°
β = 93.462 (1)°
γ = 103.972 (1)°
*V* = 908.29 (17) Å^3^
*Z* = 2
Mo *K*α radiation
μ = 1.38 mm^−1^
*T* = 298 K
0.45 × 0.41 × 0.24 mm

#### Data collection

Siemens SMART 1000 CCD diffractometer
Absorption correction: multi-scan (*SADABS*; Sheldrick, 1996 ▶) *T*_min_ = 0.575, *T*_max_ = 0.733
4646 measured reflections
3156 independent reflections
2493 reflections with *I* > 2σ(*I*)
*R*_int_ = 0.018

#### Refinement

*R*[*F* ^2^ > 2σ(*F* ^2^)] = 0.035
*wR*(*F* ^2^) = 0.096
*S* = 1.00
3156 reflections
228 parameters
H-atom parameters constrained
Δρ_max_ = 0.41 e Å^−3^
Δρ_min_ = −0.30 e Å^−3^

### 

Data collection: *SMART* (Siemens, 1996 ▶); cell refinement: *SAINT* (Siemens, 1996 ▶); data reduction: *SAINT*; program(s) used to solve structure: *SHELXS97* (Sheldrick, 2008 ▶); program(s) used to refine structure: *SHELXL97* (Sheldrick, 2008 ▶); molecular graphics: *SHELXTL* (Sheldrick, 2008 ▶); software used to prepare material for publication: *SHELXTL*.

## Supplementary Material

Crystal structure: contains datablock(s) I, global. DOI: 10.1107/S1600536811049129/bx2382sup1.cif

Structure factors: contains datablock(s) I. DOI: 10.1107/S1600536811049129/bx2382Isup2.hkl

Additional supplementary materials:  crystallographic information; 3D view; checkCIF report

## Figures and Tables

**Table 1:** Selected geometric parameters (Å, °)

Cu1—N1	1.975 (3)
Cu1—N3	2.004 (3)
Cu1—N4	2.038 (2)
Cu1—N2	2.071 (3)
Cu1—Cl1	2.4584 (10)

**Table 2:** Hydrogen-bond geometry (Å, °)

*D*—H⋯*A*	*D*—H	H⋯*A*	*D*⋯*A*	*D*—H⋯*A*
O5—H5*D*⋯Cl1	0.85	2.40	3.244 (3)	174
O5—H5*C*⋯O3^i^	0.85	2.01	2.857 (5)	173
O4—H4*D*⋯Cl1^ii^	0.85	2.44	3.275 (3)	168
O4—H4*C*⋯O1^ii^	0.85	2.60	3.165 (4)	126
O4—H4*C*⋯O1^iii^	0.85	2.23	3.063 (4)	167
O3—H3*D*⋯O5^iv^	0.85	1.94	2.785 (4)	173
O3—H3*C*⋯O4^ii^	0.85	1.96	2.802 (4)	172
O1—H1⋯O2	0.82	1.67	2.452 (4)	160

Symmetry codes: (i) 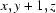; (ii) 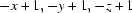; (iii) 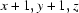; (iv) 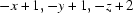.

**Fig. 1.**
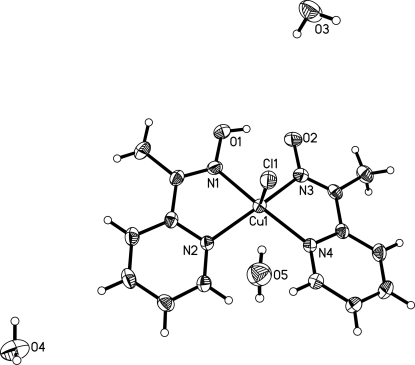
The molecular structure of the title compound with atom labels and 50% probability displacement ellipsoids for non-H atoms.

**Fig. 2.**
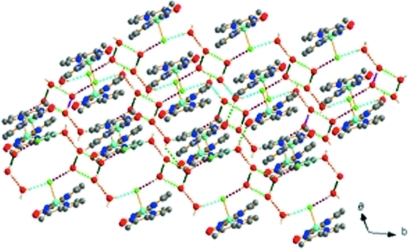
Part of the crystal structure with hydrogen bonds shown as dashed lines.

## supplementary crystallographic information

### Comment

There is currently a renewed interest in the coordination chemistry of oximes (Davidson *et al.*, 2007; Pavlishchuk *et al.*, 2003). The organic ligand is methyl 2-pyridyl ketone oxime, [(py)C(Me)NOH], which belongs to the family of 2-pyridyl oximes (Chaudhuri, 2003). 2-pyridyl oximes are a subclass of oximes whose anions are versatile ligands for a variety of research objectives and have been key ligands in several areas of molecular magnetism, including single-molecule and single-chain magnets (Clerac *et al.*, 2002). We report here the synthesis and crystal structure of the title compound. In the title complex (Fig.1) the asymmetric unit consists of one metallic complex and three water molecules. The Cu center is five-coordinate by the N atoms from the methyl(2-pyridyl)ketooxime ligand and one chloride anions.The two methyl(2-pyridyl)ketooxime ligands are coordinated to copper to form two five-membered CuC_2_N_2_ rings. The copper atom adopts a distorted 4+1 square-pyramidal coordination mode with the distortion parameter being 0.192 (Addison *et al.*, 1984) which is smaller than the values reported in the literature (Wu & Wu, 2008) and the angles around copper ion ranging from 78.86 (1)° for N1-Cu1-N2 to 168.50 (1)° for N1-Cu1-N4. There exists one deprotonated and one protonated oxime ligand with a strong intramolecular hydrogen bond between the OH group and the negatively charged oxygen of the other ligand (O1···O2 = 2.452 Å) which is shorter than the reported literature (Wu & Wu, 2008), (Table 2). The molecular conformation is stabilized by one intramolecular O—H···O and O—H···Cl hydrogen bonds interactions and the crystal structure is stabilized by six O—H···O hydrogen bonds interactions (Table 2, Fig.2).

### Experimental

A solution of CuCl_2_ (0.0426g, 0.25mmol) in MeOH (10 ml) was added to a solution of (py)C(Me)NOH (0.068 g, 0.5 mmol) in MeOH (10 ml). The resulting dark green solution was stirred for about 6 h and was then allowed to slowly concentrate by solvent evaporation at room temperature. Dark green block crystals suitable for X-ray diffraction were obtained within two weeks. (56.7%, m.p. 310-315K). FTIR (KBr) *v* (cm^-l^): 3424(O—H); 1597,(C=N); 2917, 1437, (C—H); 1157, 1177, 1260, (N—O).

### Refinement

All H atoms were placed in geometrically idealized positions (C—H 0.96(methyl), C—H 0.93(pyridyl), O—H 0.85 Å) and treated as riding on their parent atoms, with *U*_iso_(H) = 1.2*U*_eq _or 1.5*U*_eq_(C), *U*_iso_(H) =1.2*U*_eq_(O).

### Crystal data

**Table:**

[Cu(C_7_H_7_N_2_O)Cl(C_7_H_8_N_2_O)]·3H_2_O	*Z* = 2
*M**_r_* = 424.34	*F*(000) = 438
Triclinic, *P*1	*D*_x_ = 1.552 Mg m^−^^3^
Hall symbol: -P 1	Mo *K*α radiation, λ = 0.71073 Å
*a* = 8.3980 (9) Å	Cell parameters from 2075 reflections
*b* = 10.2559 (11) Å	θ = 2.5–26.8°
*c* = 12.1121 (13) Å	µ = 1.38 mm^−^^1^
α = 114.199 (2)°	*T* = 298 K
β = 93.462 (1)°	Block, dark-green
γ = 103.972 (1)°	0.45 × 0.41 × 0.24 mm
*V* = 908.29 (17) Å^3^	

### Data collection

**Table:**

Siemens SMART 1000 CCD diffractometer	3156 independent reflections
Radiation source: fine-focus sealed tube	2493 reflections with *I* > 2σ(*I*)
graphite	*R*_int_ = 0.018
phi and ω scans	θ_max_ = 25.0°, θ_min_ = 2.5°
Absorption correction: multi-scan (*SADABS*; Sheldrick, 1996)	*h* = −9→9
*T*_min_ = 0.575, *T*_max_ = 0.733	*k* = −7→12
4646 measured reflections	*l* = −14→11

### Refinement

**Table:**

Refinement on *F*^2^	Primary atom site location: structure-invariant direct methods
Least-squares matrix: full	Secondary atom site location: difference Fourier map
*R*[*F*^2^ > 2σ(*F*^2^)] = 0.035	Hydrogen site location: inferred from neighbouring sites
*wR*(*F*^2^) = 0.096	H-atom parameters constrained
*S* = 1.00	*w* = 1/[σ^2^(*F*_o_^2^) + (0.0446*P*)^2^ + 0.7087*P*] where *P* = (*F*_o_^2^ + 2*F*_c_^2^)/3
3156 reflections	(Δ/σ)_max_ = 0.022
228 parameters	Δρ_max_ = 0.41 e Å^−^^3^
0 restraints	Δρ_min_ = −0.30 e Å^−^^3^

### Fractional atomic coordinates and isotropic or equivalent isotropic displacement parameters (Å^2^)

**Table:**

	*x*	*y*	*z*	*U*_iso_*/*U*_eq_	
Cu1	0.08870 (5)	0.44895 (4)	0.68063 (3)	0.03697 (15)	
Cl1	0.35260 (11)	0.46847 (10)	0.79450 (8)	0.0481 (2)	
N1	0.0912 (4)	0.2780 (3)	0.5265 (2)	0.0418 (7)	
N2	0.1968 (3)	0.5577 (3)	0.5806 (2)	0.0364 (6)	
N3	−0.0916 (3)	0.3230 (3)	0.7265 (3)	0.0403 (7)	
N4	0.0355 (3)	0.6084 (3)	0.8295 (2)	0.0355 (6)	
O1	0.0222 (3)	0.1363 (2)	0.5044 (2)	0.0568 (7)	
H1	−0.0247	0.1338	0.5613	0.085*	
O2	−0.1472 (3)	0.1713 (3)	0.6666 (2)	0.0555 (7)	
O3	0.4005 (4)	0.0145 (3)	0.8601 (3)	0.0820 (10)	
H3C	0.3588	0.0494	0.8165	0.098*	
H3D	0.4073	0.0722	0.9355	0.098*	
O4	0.7339 (4)	0.8940 (3)	0.3036 (3)	0.0840 (10)	
H4C	0.8180	0.9495	0.3605	0.101*	
H4D	0.7251	0.8032	0.2868	0.101*	
O5	0.5675 (4)	0.8149 (3)	0.8877 (3)	0.0795 (9)	
H5C	0.5105	0.8684	0.8772	0.095*	
H5D	0.5043	0.7260	0.8616	0.095*	
C1	0.1862 (6)	0.1812 (4)	0.3309 (3)	0.0639 (11)	
H1A	0.2069	0.1028	0.3481	0.096*	
H1B	0.2772	0.2196	0.2977	0.096*	
H1C	0.0847	0.1424	0.2722	0.096*	
C2	0.1705 (4)	0.3039 (4)	0.4468 (3)	0.0421 (8)	
C3	0.2392 (4)	0.4625 (4)	0.4784 (3)	0.0373 (7)	
C4	0.3404 (4)	0.5153 (4)	0.4109 (3)	0.0474 (9)	
H4	0.3695	0.4487	0.3416	0.057*	
C5	0.3981 (5)	0.6664 (5)	0.4465 (3)	0.0534 (10)	
H5	0.4678	0.7030	0.4024	0.064*	
C6	0.3516 (5)	0.7628 (4)	0.5479 (3)	0.0506 (9)	
H6	0.3880	0.8655	0.5732	0.061*	
C7	0.2498 (4)	0.7036 (4)	0.6111 (3)	0.0439 (8)	
H7	0.2163	0.7687	0.6786	0.053*	
C8	−0.3054 (5)	0.3094 (4)	0.8534 (4)	0.0558 (10)	
H8A	−0.3315	0.2037	0.8035	0.084*	
H8B	−0.4012	0.3419	0.8436	0.084*	
H8C	−0.2750	0.3313	0.9382	0.084*	
C9	−0.1636 (4)	0.3891 (4)	0.8147 (3)	0.0397 (8)	
C10	−0.0901 (4)	0.5520 (4)	0.8775 (3)	0.0375 (7)	
C11	−0.1392 (5)	0.6427 (4)	0.9816 (3)	0.0502 (9)	
H11	−0.2280	0.6026	1.0116	0.060*	
C12	−0.0552 (5)	0.7932 (4)	1.0402 (4)	0.0577 (10)	
H12	−0.0871	0.8559	1.1100	0.069*	
C13	0.0756 (5)	0.8495 (4)	0.9948 (3)	0.0505 (9)	
H13	0.1353	0.9503	1.0339	0.061*	
C14	0.1169 (4)	0.7534 (4)	0.8898 (3)	0.0436 (8)	
H14	0.2063	0.7920	0.8595	0.052*	

### Atomic displacement parameters (Å^2^)

**Table:**

	*U*^11^	*U*^22^	*U*^33^	*U*^12^	*U*^13^	*U*^23^
Cu1	0.0435 (3)	0.0338 (2)	0.0336 (2)	0.01245 (17)	0.01058 (18)	0.01352 (17)
Cl1	0.0494 (5)	0.0579 (6)	0.0446 (5)	0.0244 (4)	0.0113 (4)	0.0246 (4)
N1	0.0512 (18)	0.0330 (15)	0.0382 (16)	0.0145 (13)	0.0054 (14)	0.0118 (12)
N2	0.0428 (16)	0.0390 (15)	0.0312 (14)	0.0154 (12)	0.0093 (12)	0.0170 (12)
N3	0.0412 (16)	0.0367 (15)	0.0424 (16)	0.0098 (12)	0.0051 (13)	0.0180 (13)
N4	0.0402 (16)	0.0366 (15)	0.0340 (14)	0.0148 (12)	0.0107 (12)	0.0170 (12)
O1	0.0778 (19)	0.0337 (13)	0.0522 (15)	0.0157 (12)	0.0182 (14)	0.0119 (12)
O2	0.0658 (17)	0.0346 (13)	0.0603 (16)	0.0062 (12)	0.0103 (14)	0.0201 (12)
O3	0.113 (3)	0.0569 (18)	0.070 (2)	0.0055 (17)	0.0038 (19)	0.0344 (16)
O4	0.097 (3)	0.0676 (19)	0.074 (2)	0.0333 (18)	−0.0174 (18)	0.0177 (17)
O5	0.075 (2)	0.073 (2)	0.087 (2)	0.0174 (17)	0.0154 (18)	0.0332 (18)
C1	0.088 (3)	0.052 (2)	0.043 (2)	0.030 (2)	0.020 (2)	0.0075 (18)
C2	0.049 (2)	0.046 (2)	0.0309 (17)	0.0222 (16)	0.0064 (16)	0.0121 (15)
C3	0.0375 (19)	0.052 (2)	0.0283 (16)	0.0204 (15)	0.0055 (14)	0.0187 (15)
C4	0.051 (2)	0.064 (2)	0.0372 (19)	0.0252 (19)	0.0133 (17)	0.0253 (18)
C5	0.054 (2)	0.076 (3)	0.048 (2)	0.023 (2)	0.0184 (19)	0.042 (2)
C6	0.060 (2)	0.051 (2)	0.046 (2)	0.0140 (18)	0.0075 (18)	0.0272 (18)
C7	0.053 (2)	0.046 (2)	0.0337 (18)	0.0160 (17)	0.0093 (16)	0.0178 (16)
C8	0.044 (2)	0.068 (3)	0.067 (3)	0.0134 (19)	0.0167 (19)	0.041 (2)
C9	0.0348 (18)	0.050 (2)	0.0443 (19)	0.0130 (15)	0.0057 (15)	0.0302 (17)
C10	0.0376 (19)	0.0488 (19)	0.0365 (18)	0.0196 (15)	0.0086 (15)	0.0245 (16)
C11	0.051 (2)	0.065 (2)	0.049 (2)	0.0258 (19)	0.0245 (18)	0.0308 (19)
C12	0.071 (3)	0.058 (2)	0.047 (2)	0.032 (2)	0.027 (2)	0.0164 (19)
C13	0.065 (3)	0.043 (2)	0.044 (2)	0.0227 (18)	0.0137 (19)	0.0157 (17)
C14	0.049 (2)	0.0412 (19)	0.0411 (19)	0.0136 (16)	0.0113 (16)	0.0175 (16)

### Geometric parameters (Å, °)

**Table:**

Cu1—N1	1.975 (3)	C1—H1C	0.9600
Cu1—N3	2.004 (3)	C2—C3	1.462 (5)
Cu1—N4	2.038 (2)	C3—C4	1.382 (5)
Cu1—N2	2.071 (3)	C4—C5	1.374 (5)
Cu1—Cl1	2.4584 (10)	C4—H4	0.9300
N1—C2	1.284 (4)	C5—C6	1.374 (5)
N1—O1	1.333 (3)	C5—H5	0.9300
N2—C7	1.333 (4)	C6—C7	1.377 (5)
N2—C3	1.357 (4)	C6—H6	0.9300
N3—C9	1.284 (4)	C7—H7	0.9300
N3—O2	1.359 (3)	C8—C9	1.490 (5)
N4—C14	1.331 (4)	C8—H8A	0.9600
N4—C10	1.357 (4)	C8—H8B	0.9600
O1—H1	0.8200	C8—H8C	0.9600
O3—H3C	0.8500	C9—C10	1.467 (5)
O3—H3D	0.8499	C10—C11	1.382 (5)
O4—H4C	0.8500	C11—C12	1.379 (5)
O4—H4D	0.8499	C11—H11	0.9300
O5—H5C	0.8501	C12—C13	1.367 (5)
O5—H5D	0.8500	C12—H12	0.9300
C1—C2	1.492 (4)	C13—C14	1.379 (5)
C1—H1A	0.9600	C13—H13	0.9300
C1—H1B	0.9600	C14—H14	0.9300
			
N1—Cu1—N3	92.87 (11)	C4—C3—C2	123.6 (3)
N1—Cu1—N4	168.50 (11)	C5—C4—C3	119.8 (3)
N3—Cu1—N4	78.92 (10)	C5—C4—H4	120.1
N1—Cu1—N2	78.86 (11)	C3—C4—H4	120.1
N3—Cu1—N2	157.52 (11)	C4—C5—C6	119.3 (3)
N4—Cu1—N2	105.76 (10)	C4—C5—H5	120.4
N1—Cu1—Cl1	96.51 (9)	C6—C5—H5	120.4
N3—Cu1—Cl1	105.86 (8)	C5—C6—C7	118.3 (3)
N4—Cu1—Cl1	93.50 (8)	C5—C6—H6	120.8
N2—Cu1—Cl1	95.89 (8)	C7—C6—H6	120.8
C2—N1—O1	118.3 (3)	N2—C7—C6	123.4 (3)
C2—N1—Cu1	118.7 (2)	N2—C7—H7	118.3
O1—N1—Cu1	122.9 (2)	C6—C7—H7	118.3
C7—N2—C3	118.0 (3)	C9—C8—H8A	109.5
C7—N2—Cu1	129.7 (2)	C9—C8—H8B	109.5
C3—N2—Cu1	111.4 (2)	H8A—C8—H8B	109.5
C9—N3—O2	117.7 (3)	C9—C8—H8C	109.5
C9—N3—Cu1	118.3 (2)	H8A—C8—H8C	109.5
O2—N3—Cu1	123.9 (2)	H8B—C8—H8C	109.5
C14—N4—C10	117.7 (3)	N3—C9—C10	113.8 (3)
C14—N4—Cu1	128.6 (2)	N3—C9—C8	123.8 (3)
C10—N4—Cu1	113.4 (2)	C10—C9—C8	122.3 (3)
N1—O1—H1	109.5	N4—C10—C11	121.6 (3)
H3C—O3—H3D	108.6	N4—C10—C9	115.3 (3)
H4C—O4—H4D	108.7	C11—C10—C9	123.0 (3)
H5C—O5—H5D	108.6	C12—C11—C10	119.3 (3)
C2—C1—H1A	109.5	C12—C11—H11	120.4
C2—C1—H1B	109.5	C10—C11—H11	120.4
H1A—C1—H1B	109.5	C13—C12—C11	119.3 (3)
C2—C1—H1C	109.5	C13—C12—H12	120.3
H1A—C1—H1C	109.5	C11—C12—H12	120.3
H1B—C1—H1C	109.5	C12—C13—C14	118.5 (3)
N1—C2—C3	114.2 (3)	C12—C13—H13	120.7
N1—C2—C1	122.3 (3)	C14—C13—H13	120.7
C3—C2—C1	123.5 (3)	N4—C14—C13	123.5 (3)
N2—C3—C4	121.1 (3)	N4—C14—H14	118.2
N2—C3—C2	115.3 (3)	C13—C14—H14	118.2
			
N3—Cu1—N1—C2	167.8 (3)	Cu1—N1—C2—C1	176.8 (3)
N4—Cu1—N1—C2	123.8 (5)	C7—N2—C3—C4	2.8 (5)
N2—Cu1—N1—C2	8.9 (3)	Cu1—N2—C3—C4	−167.7 (3)
Cl1—Cu1—N1—C2	−85.9 (3)	C7—N2—C3—C2	−177.2 (3)
N3—Cu1—N1—O1	−16.2 (3)	Cu1—N2—C3—C2	12.3 (3)
N4—Cu1—N1—O1	−60.2 (7)	N1—C2—C3—N2	−5.6 (4)
N2—Cu1—N1—O1	−175.1 (3)	C1—C2—C3—N2	172.9 (3)
Cl1—Cu1—N1—O1	90.2 (3)	N1—C2—C3—C4	174.4 (3)
N1—Cu1—N2—C7	179.7 (3)	C1—C2—C3—C4	−7.2 (5)
N3—Cu1—N2—C7	109.7 (4)	N2—C3—C4—C5	−0.8 (5)
N4—Cu1—N2—C7	10.5 (3)	C2—C3—C4—C5	179.3 (3)
Cl1—Cu1—N2—C7	−84.8 (3)	C3—C4—C5—C6	−1.1 (6)
N1—Cu1—N2—C3	−11.2 (2)	C4—C5—C6—C7	0.8 (6)
N3—Cu1—N2—C3	−81.2 (3)	C3—N2—C7—C6	−3.2 (5)
N4—Cu1—N2—C3	179.6 (2)	Cu1—N2—C7—C6	165.3 (3)
Cl1—Cu1—N2—C3	84.3 (2)	C5—C6—C7—N2	1.5 (6)
N1—Cu1—N3—C9	−167.7 (3)	O2—N3—C9—C10	177.3 (3)
N4—Cu1—N3—C9	4.2 (2)	Cu1—N3—C9—C10	−5.3 (4)
N2—Cu1—N3—C9	−100.3 (3)	O2—N3—C9—C8	−0.9 (5)
Cl1—Cu1—N3—C9	94.7 (2)	Cu1—N3—C9—C8	176.5 (3)
N1—Cu1—N3—O2	9.6 (3)	C14—N4—C10—C11	3.7 (5)
N4—Cu1—N3—O2	−178.5 (3)	Cu1—N4—C10—C11	177.5 (3)
N2—Cu1—N3—O2	76.9 (4)	C14—N4—C10—C9	−173.8 (3)
Cl1—Cu1—N3—O2	−88.0 (2)	Cu1—N4—C10—C9	0.0 (3)
N1—Cu1—N4—C14	−144.0 (5)	N3—C9—C10—N4	3.4 (4)
N3—Cu1—N4—C14	171.0 (3)	C8—C9—C10—N4	−178.4 (3)
N2—Cu1—N4—C14	−31.6 (3)	N3—C9—C10—C11	−174.1 (3)
Cl1—Cu1—N4—C14	65.5 (3)	C8—C9—C10—C11	4.1 (5)
N1—Cu1—N4—C10	43.0 (6)	N4—C10—C11—C12	−2.2 (5)
N3—Cu1—N4—C10	−2.0 (2)	C9—C10—C11—C12	175.2 (3)
N2—Cu1—N4—C10	155.4 (2)	C10—C11—C12—C13	−0.4 (6)
Cl1—Cu1—N4—C10	−107.5 (2)	C11—C12—C13—C14	1.3 (6)
O1—N1—C2—C3	179.1 (3)	C10—N4—C14—C13	−2.8 (5)
Cu1—N1—C2—C3	−4.7 (4)	Cu1—N4—C14—C13	−175.6 (3)
O1—N1—C2—C1	0.6 (5)	C12—C13—C14—N4	0.4 (6)

### Hydrogen-bond geometry (Å, °)

**Table:**

*D*—H···*A*	*D*—H	H···*A*	*D*···*A*	*D*—H···*A*
O5—H5D···Cl1	0.85	2.40	3.244 (3)	174.
O5—H5C···O3^i^	0.85	2.01	2.857 (5)	173.
O4—H4D···Cl1^ii^	0.85	2.44	3.275 (3)	168.
O4—H4C···O1^ii^	0.85	2.60	3.165 (4)	126.
O4—H4C···O1^iii^	0.85	2.23	3.063 (4)	167.
O3—H3D···O5^iv^	0.85	1.94	2.785 (4)	173.
O3—H3C···O4^ii^	0.85	1.96	2.802 (4)	172.
O1—H1···O2	0.82	1.67	2.452 (4)	160.

Symmetry codes: (i) *x*, *y*+1, *z*; (ii) −*x*+1, −*y*+1, −*z*+1; (iii) *x*+1, *y*+1, *z*; (iv) −*x*+1, −*y*+1, −*z*+2.
